# Error rate for imputation from the Illumina BovineSNP50 chip to the Illumina BovineHD chip

**DOI:** 10.1186/1297-9686-46-10

**Published:** 2014-02-04

**Authors:** Chris Schrooten, Romain Dassonneville, Vincent Ducrocq, Rasmus F Brøndum, Mogens S Lund, Jun Chen, Zengting Liu, Oscar González-Recio, Juan Pena, Tom Druet

**Affiliations:** 1 CRV BV, P.O. Box 454, 6800 AL Arnhem, The Netherlands; 2 INRA UMR1313 Génétique Animale et Biologie Intégrative, Jouy-en-Josas F-78350, France; 3 AgroParisTech, UMR1313 Génétique Animale et Biologie Intégrative, Paris F-75231, France; 4 Institut de l’Elevage, 149 rue de Bercy, Paris 75595, France; 5 Department of Molecular Biology and Genetics, Aarhus University, Tjele, Aarhus DK-8830, Denmark; 6 VIT, Heideweg 1, Verden 27283, Germany; 7 INIA, Ctra La Coruña km 7.5, 28040 Madrid, Spain; 8 CONAFE- Confederación de Asociaciones de Frisona Española, Ctra. de Andalucía, km. 23,600, Valdemoro, 28340 Madrid Spain; 9 Unit of Animal Genomics, Faculty of Veterinary Medicine and Centre for Biomedical Integrative Genoproteinomics, University of Liège, Liège B-4000, Belgium

## Abstract

**Background:**

Imputation of genotypes from low-density to higher density chips is a cost-effective method to obtain high-density genotypes for many animals, based on genotypes of only a relatively small subset of animals (reference population) on the high-density chip. Several factors influence the accuracy of imputation and our objective was to investigate the effects of the size of the reference population used for imputation and of the imputation method used and its parameters. Imputation of genotypes was carried out from 50 000 (moderate-density) to 777 000 (high-density) SNPs (single nucleotide polymorphisms).

**Methods:**

The effect of reference population size was studied in two datasets: one with 548 and one with 1289 Holstein animals, genotyped with the Illumina BovineHD chip (777 k SNPs). A third dataset included the 548 animals genotyped with the 777 k SNP chip and 2200 animals genotyped with the Illumina BovineSNP50 chip. In each dataset, 60 animals were chosen as validation animals, for which all high-density genotypes were masked, except for the Illumina BovineSNP50 markers. Imputation was studied in a subset of six chromosomes, using the imputation software programs Beagle and DAGPHASE.

**Results:**

Imputation with DAGPHASE and Beagle resulted in 1.91% and 0.87% allelic imputation error rates in the dataset with 548 high-density genotypes, when scale and shift parameters were 2.0 and 0.1, and 1.0 and 0.0, respectively. When Beagle was used alone, the imputation error rate was 0.67%. If the information obtained by Beagle was subsequently used in DAGPHASE, imputation error rates were slightly higher (0.71%). When 2200 moderate-density genotypes were added and Beagle was used alone, imputation error rates were slightly lower (0.64%). The least imputation errors were obtained with Beagle in the reference set with 1289 high-density genotypes (0.41%).

**Conclusions:**

For imputation of genotypes from the 50 k to the 777 k SNP chip, Beagle gave the lowest allelic imputation error rates. Imputation error rates decreased with increasing size of the reference population. For applications for which computing time is limiting, DAGPHASE using information from Beagle can be considered as an alternative, since it reduces computation time and increases imputation error rates only slightly.

## Background

Since 2007, large numbers of dairy cattle have been genotyped with various 50 k chips, mainly the BovineSNP50 BeadChip [1]. Genotypes obtained from these chips can be used to perform association studies to identify loci that affect traits of interest, or to obtain more reliable breeding values at a younger age, to enable genomic selection of animals. These genomically enhanced breeding values (GEBV) are used routinely in several breeding programs. Apart from the BovineSNP50 BeadChip, other 50 k chips have been developed. Genotype imputation, which predicts marker genotypes at non-genotyped loci, has been used to share information generated by different 50 k chips, and facilitates the exchange of genotypes between organizations using different chips [2,3]. Imputation uses either population-based or family-based linkage disequilibrium (LD) between loci, or a combination of these two types of information, to derive genotypes at non-genotyped loci. Various imputation algorithms and software have been developed. The most commonly used software packages for bovine datasets are Beagle [4], AlphaImpute [5], Fimpute [6], Impute [7], findhap [8] and the PHASEBOOK package, which consists of LinkPHASE and DAGPHASE [9]. Imputation has been shown to be accurate: imputation between two different 50 k chips with approximately 10 k SNPs in common resulted in an allelic imputation error rate of 1.0% when using DAGPHASE, and when approximately 1000 animals had genotypes on both chips [2]. A 1.0% imputation error rate is considered sufficiently low to allow exchange of genotypes between different SNP chips [2].

Imputation can also be applied to predict genotypes on higher density chips from lower density chips, e.g. the Illumina Bovine3K chip [10], that contains 2.9 k SNPs, or the Illumina BovineLD chip [11,12], with 6.9 k SNPs. Several authors have investigated imputation error rates obtained when genotypes were imputed from this or other low-density panels to the Illumina BovineSNP50 chips. Weigel et al. [13] used a Jersey reference population of 2542 animals genotyped with 43 k SNPs. They applied IMPUTE 2.0 [7] and found genotype imputation error rates of 10.8% and 5.8% when validation animals had genotypes for only 5 or 10% of the SNPs, respectively. Thus, allelic imputation error rates were approximately equal to 5.4 and 2.9%, respectively. Dassonneville et al. [14] found imputation error rates of 5.5% and 3.9% in a Nordic and French dataset with approximately 3000 reference animals, when imputation was performed from the Illumina Bovine3K chip to the Illumina Bovine50K chip. When the size of the reference set was approximately 11 000 (dataset 1) or 12 000 animals (dataset 2), imputation error rates were 4.0 and 2.1%, respectively. Mulder et al. [15] found allelic imputation error rates of 3.8 and 2.8% when imputing from an in-silico chip of 3 k and 6 k SNPs, respectively, to 50 k SNPs, using DAGPHASE [9] and a reference set for imputation of approximately 5300 animals. Dassonneville et al. [16] studied imputation error rates using the Beagle software [4] for imputing genotypes from the Illumina Bovine 3 K and two in-silico chips (with 3 k and 6 k SNPs) to the BovineSNP50 chip in the Holstein, Montbéliarde, and Blonde d’Aquitaine breeds. Within breeds, the lowest allelic imputation error rate was 0.9% for imputation from the 6 k in-silico chip with the Holstein breed and the highest error rate was 4.8% for imputation from the Illumina Bovine3K chip with the Blonde d’Aquitaine breed. Differences between breeds were to a large extent due to the size of the reference population in each breed. The reference population for imputation was largest in Holstein (3071 animals) and lowest in Blonde d’Aquitaine (754 animals).

In 2010, the Illumina BovineHD BeadChip [17] with approximately 777 k SNPs became available. A strategy to take advantage of the BovineHD chip could be to re-genotype all animals previously genotyped with low- or moderate-density chips, but the resulting gain in reliability of GEBV may not outweigh the large additional cost. Therefore, genotyping part of the population with the BovineHD chip and subsequently imputing the animals genotyped with 50 k chips to the HD chip is considered an attractive alternative, provided that imputation can be done with a low error rate.

Imputation error rate depends on several factors, some of which were illustrated in the above examples: (1) effective population size; (2) number of markers on the lower density and the higher density chips; (3) distribution of markers on those chips; (4) (minor) allele frequency of the imputed alleles; (5) quality of the marker map used for imputation; (6) relationship between the animals in the reference set and the animals to impute; (7) number of animals in the reference set for imputation (i.e. genotyped on high density); and (8) the imputation method and, if applicable, the parameter settings used. Factors 6–8 are to some extent under our control and can be varied to study their effect on imputation error rates. The objective of this study was to investigate the effect of reference population size used and the imputation method and its parameters on the error rate for imputation from the BovineSNP50 to the BovineHD chip.

## Methods

### Genotypes and animals

Datasets composed of animals genotyped with the Illumina BovineHD chip (Illumina, San Diego, California, USA) were created. In the remaining of this manuscript, this chip will be referred to as the HD-chip. Dataset 1 (Table 1) consisted of the first batch of animals genotyped with the HD-chip by the Eurogenomics consortium. These were 548 high impact bulls from the Eurogenomics reference population [3], for which the call rate (fraction of SNPs for which the genotype was scored) was at least 0.90. Dataset 2 consisted of the animals in dataset 1, plus 410 males and 331 females. All 1289 animals had a call rate of 0.90 or higher. In dataset 1, four different validation groups were formed, each consisting of 60 different animals that did not have any descendants with HD-genotypes. For the validation animals, the genotypes of all markers except those on the Illumina BovineSNP50 [1] were masked. In the remaining of this manuscript, this chip will be referred to as the 50 k-chip. The HD-genotypes of the remaining 488 animals in each subset were used as reference to impute the masked genotypes of the 60 validation animals. In dataset 2, the same four validation groups were formed as in dataset 1, and the reference set consisted of the remaining 1229 animals.

**Table 1:** Number and type of animals in each of the three analyzed datasets

**Dataset**	**HD reference**		**Additional 50 k genotypes (males)**	**Validation animals (males)**	**Imputation method applied**			
	**Males**	**Females**			**A**	**B**	**C**	**D**
					**Beagle 2.1.3/DAGPHASE**	**Beagle 2.1.3/DAGPHASE**	**Beagle 3.3.0**	**DAGPHASE**
					**scale 2.0, shift 0.1**	**scale 1.0, shift 0.0**		
Dataset 1	488	0	0	60	Yes	Yes	Yes	Yes
Dataset 2	898	331	0	60	No	No	Yes	No
Dataset 3	488	0	2200	60	Yes	No	Yes	No

In addition to these two datasets that contained only animals with HD genotypes, a third dataset was constituted as an alternative to dataset 1. In dataset 3, 2200 bulls genotyped with the 50 k-chip were added, to study the effect on imputation of adding information that could assist in optimally phasing the HD genotypes.

Genotype data was edited as follows: (1) SNPs with a minor allele frequency less than 0.025 were removed; (2) Hardy-Weinberg equilibrium: SNPs for which the fraction of heterozygotes deviated by more than 0.15 from the expected fraction of heterozygotes based on allele frequencies were removed; (3) call rate: SNPs with called genotypes for less than 90% of the animals were removed.

In all datasets, only SNPs mapped to either BTA1, 6, 11, 14, 20, or 29 were selected. This subset was chosen to reduce computational effort, and this number of chromosomes was considered large enough to produce imputation results that would be representative for the whole genome, and also provide insight into variation between chromosomes with regard to imputation results. Details on the chromosomes used in the analysis, with regard to the number of unmasked loci, total number of loci, fraction of unmasked loci in validation animals, and length of the chromosome are in Table 2.

**Table 2:** Details on chromosomes in the analyses

**BTA**	**n_50k**	**n_HD**	**fr_unm**	**Length(cM)**	**n50k/cM**
1	2571	37791	0.0680	158.32	16.24
6	1989	29805	0.0667	119.45	16.65
11	1696	27398	0.0619	107.28	15.81
14	1376	17269	0.0797	84.63	16.26
20	1164	18381	0.0633	71.99	16.17
29	793	12690	0.0625	51.50	15.40

For each chromosome: number of markers that were not masked in validation animals (n_50k), total number of markers (n_HD), fraction unmasked (fr_unm), length of chromosome in centiMorgan, and average number of unmasked markers per cM (n50k/cM).

### Imputation

The marker map used for imputation was the UMD 3.1 map (University of Maryland, College Park, MD). In dataset 1, imputation was performed using the different imputation software packages with parameter values presented in Table 1. Method A was the method described by Druet and Georges [9], and uses both linkage and linkage disequilibrium information to optimize phasing and imputation. Briefly this method consisted of phasing haplotypes partially using LinkPhase [9], 10 iterative rounds of DAGPHASE [9] and Beagle 2.1.3 [4] to optimize phasing in the reference animals, and 10 rounds of alternating DAGPHASE and Beagle on the complete dataset, followed by one round of DAGPHASE using the Viterbi algorithm [18] for haplotype reconstruction and imputation. The scale and shift parameters for Beagle were equal to 2.0 and 0.1, respectively. These parameters determine if clusters of haplotypes share enough similarity to merge into one cluster or not. The number of remaining clusters increases with decreasing values of the scale and shift parameters, and imputation accuracy is higher with a higher number of remaining clusters. Full details of this method can be found in [9]. Method B was the same as method A, except for the scale and shift parameters, which were chosen to be equal to 1.0 and 0.0. These are the default values used in Beagle. In method C, phasing and imputation were performed with Beagle (version 3.3.0), without using pedigree (linkage) information. In Beagle 3.3.0, the default values for scale and shift are equal to 1.0 and 0.0, and cannot be changed. Method D used the directed acyclic graph (DAG) generated by Beagle in method C as input for one round of DAGPHASE. This method was considered as an attractive alternative to method C, because the already available DAG can be used for new animals genotyped on the moderate-density chip, without the need to impute previously imputed animals again [16].

The effect of imputation software and its parameters on imputation errors was obtained from analysis of dataset 1. The results of method C in datasets 1 and 2 provide insight on the effect of reference population size on imputation error rates. The effect of adding animals genotyped at medium density on imputation error rates is obtained by comparing results from dataset 1 and dataset 3.

### Evaluation

Datasets and imputation methods were evaluated for their allelic imputation error rate:

(1) $$\mathrm{err}\left(\%\right)=\frac{n_{\mathrm{imputed}\neq \mathrm{observed}}}{n_{\mathrm{imputed}.\mathrm{and}.\mathrm{observed}}}*100,$$

where err(%) = allelic imputation error rate, as a percentage; n_imputed≠observed_ = number of alleles for which the imputed allele was not equal to the non-missing observed allele; n_imputed.and.observed_ = number of alleles for which the imputed allele was compared with the non-missing observed allele.

More information on imputation error rates was obtained by calculating the average allelic imputation error rate for a number of traceability classes. Traceability is defined as the additive relationship of a validation animal with its closest HD-genotyped ancestors, summed across all ancestry paths [15]. This can also be interpreted as the expected proportion of the genome inherited from HD-genotyped ancestors. Traceability has values between 0 and 1 when not accounting for inbreeding in the population. Validation animals were assigned to classes with 0.025 difference between highest and lowest traceability. Adjacent classes were joined into one class, to reduce the number of classes and to have approximately the same number of animals in all classes. The difference between highest and lowest traceability value in each class was maximized at 0.25. For each class, the average imputation error rate was computed.

Higher imputation error rates are expected for low frequency alleles, because the reference population may contain limited or no information on the haplotypes that contain these alleles. To verify this, allelic imputation error rates will be presented as a function of the allele frequency, on a per allele basis. Because of the low frequency alleles, the contribution to overall imputation error rate per locus may be limited, and therefore the imputation error rate will also be presented as a function of the minor allele frequency. This is evaluated on a per locus basis, with imputation errors of both the low frequency and the high frequency allele being combined.

## Results

Table 3 shows average imputation error rates across four replicates in the analyzed datasets and models. In dataset 1, with 488 reference animals genotyped with the BovineHD chip, Beagle and DAGPHASE with scale and shift parameters equal to 2.0 and 0.1 (method A) resulted in an average imputation error rate of 1.91% for the six analyzed chromosomes. When scale and shift parameters were changed to 1.0 and 0.0 respectively (method B), the imputation error rate decreased to 0.87%. With Beagle alone (method C), the imputation error rate was 0.67%. When the information from method C was used in one final round of DAGPHASE (method D), a slightly higher imputation error rate (0.71%) was found. Methods A and C were also applied to dataset 3, which comprised 2200 additional animals with BovineSNP50 genotypes. This resulted in 0.03-0.04% lower imputation error rates than obtained without the additional 2200 BovineSNP50 genotypes. Method C was also applied to a dataset consisting of 1229 animals genotyped with the BovineHD chip (dataset 2). The average imputation error rate in dataset 2 was 0.41%, which was lower than what was obtained with method C in dataset 1 (0.67%) and in dataset 3 (0.64%).

**Table 3:** Average allelic imputation error rate (%) for each of six chromosomes and averaged across chromosomes

**BTA**	**Dataset/Method**						
	**Dataset 1**				**Dataset 2**	**Dataset 3**	
	**A**	**B**	**C**	**D**	**C**	**A**	**C**
1	1.82	0.89	0.72	0.76	0.47	1.80	0.70
6	1.73	0.81	0.61	0.65	0.38	1.71	0.58
11	2.05	0.89	0.66	0.71	0.40	1.98	0.61
14	1.82	0.76	0.56	0.61	0.36	1.76	0.54
20	1.94	0.86	0.68	0.71	0.35	1.88	0.64
29	2.41	1.05	0.82	0.84	0.52	2.38	0.78
Average	1.91	0.87	0.67	0.71	0.41	1.87	0.64

Results are presented for four methods and three datasets: method A is a combination of Beagle 2.1.3 and DAGPHASE with scale and shift parameters equal to 2.0 and 0.1; method B is the same as method A, but with scale and shift parameters equal to 1.0 and 0.0; method C is Beagle version 3.3.0; method D is DAGPHASE using DAG from method C.

There were large differences in allelic imputation error rates between chromosomes (Table 3). The lowest imputation error rate was observed for BTA14 in most dataset-method combinations, except for method A, where BTA6 had the lowest imputation error rate. In all analyses, the imputation error rate was highest for BTA29.

**Figure 1:**
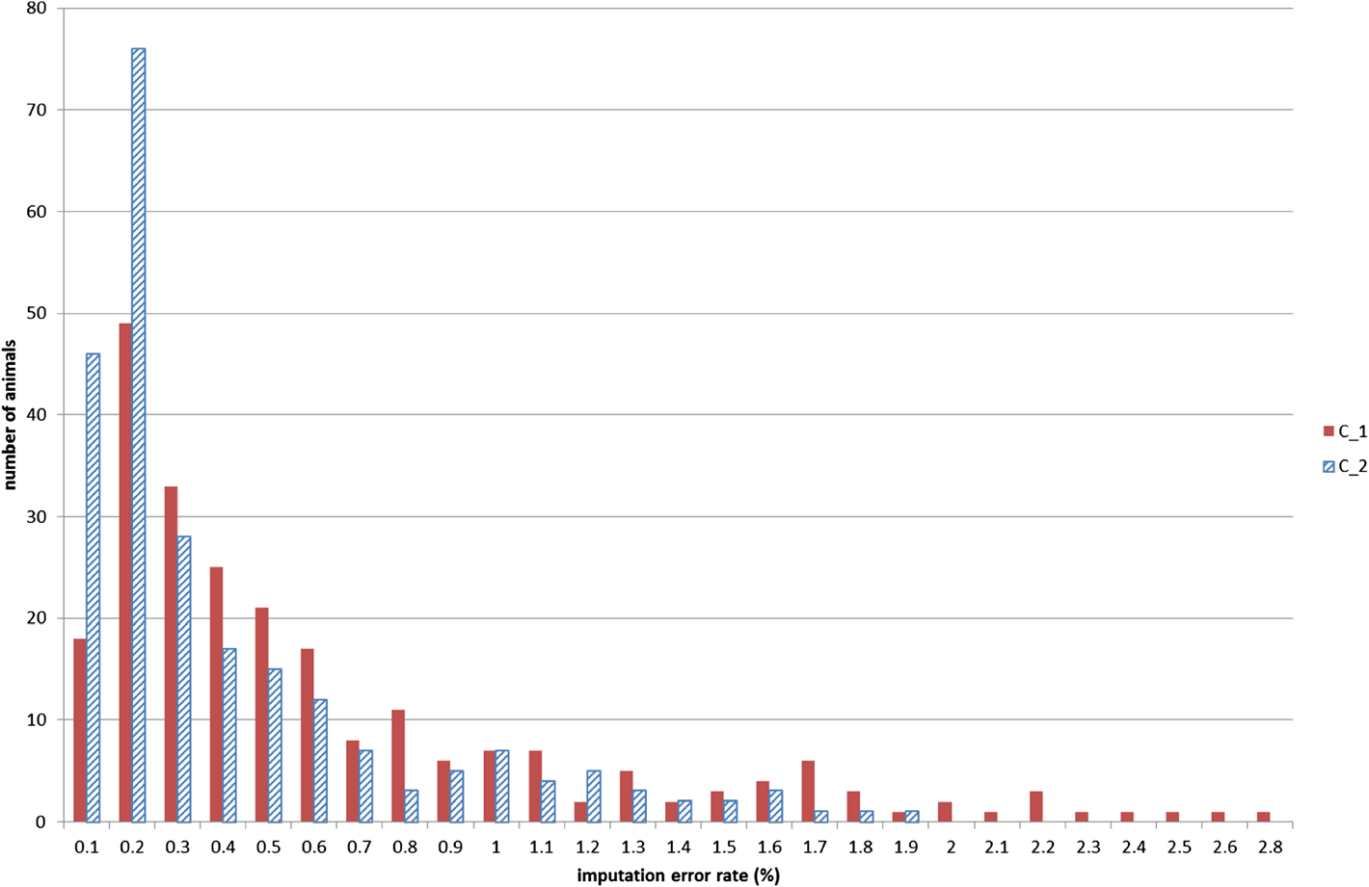
Number of validation animals per imputation error rate class in dataset 1 (C_1) and dataset 2 (C_2), using method C (Beagle version 3.3.0).

Figure 1 shows the number of validation animals per class of imputation error rate, for method C and datasets 1 and 2. The distribution was skewed, with the largest number of animals in the lower imputation error rate classes.

**Figure 2:**
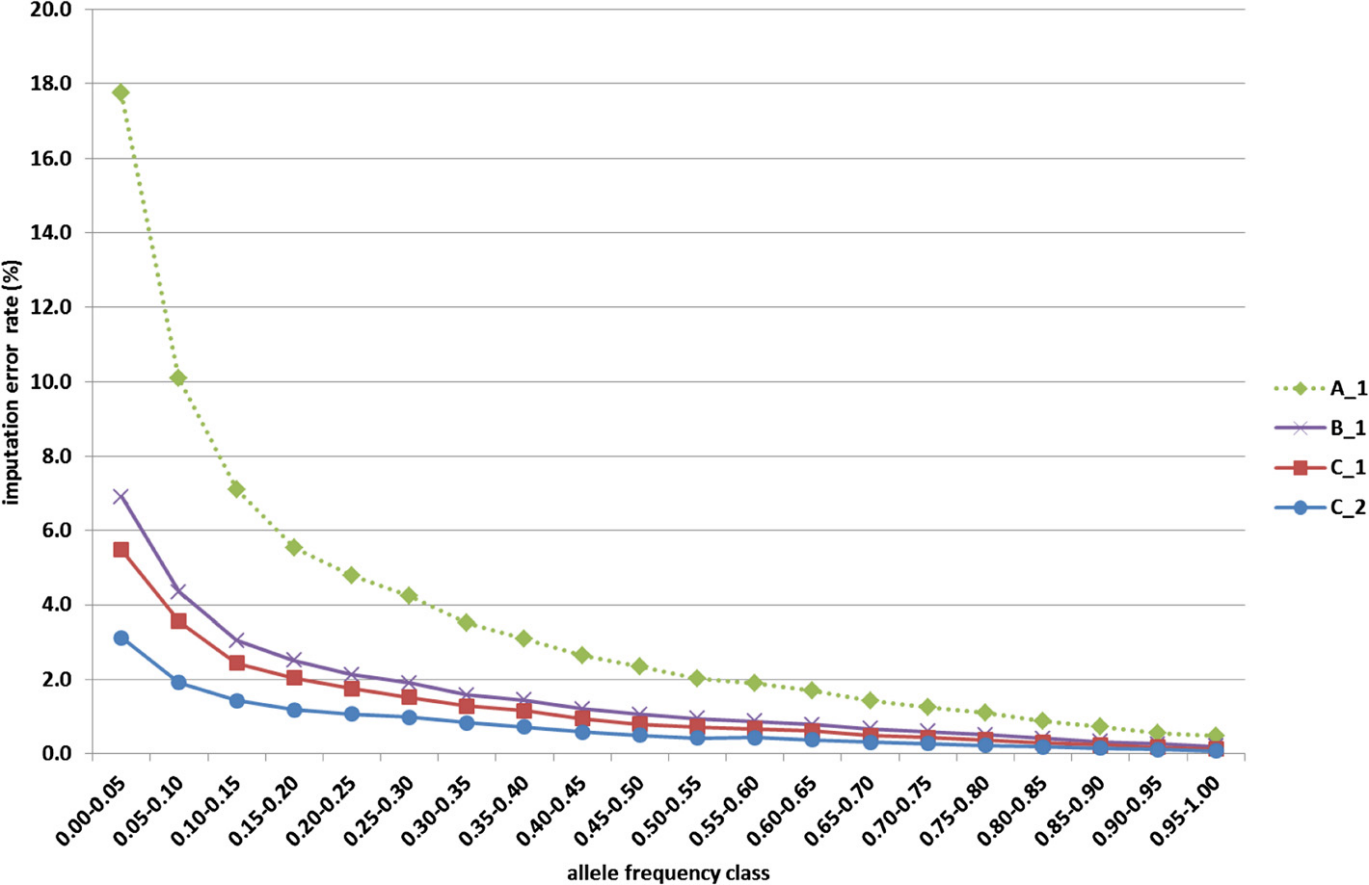
**Average allelic imputation error rate (%) for each of 20 classes of allele frequency.** Results are presented for four combinations of method (A, B or C) and dataset (1 or 2); method A: combination of Beagle 2.1.3 and DAGPHASE with scale and shift parameters equal to 2.0 and 0.1; method B: same as method A, but with scale and shift parameters equal to 1.0 and 0.0; method C: Beagle version 3.3.0; dataset 1: 548 HD-genotyped animals; dataset 2: 1289 HD-genotyped animals.

**Figure 3:**
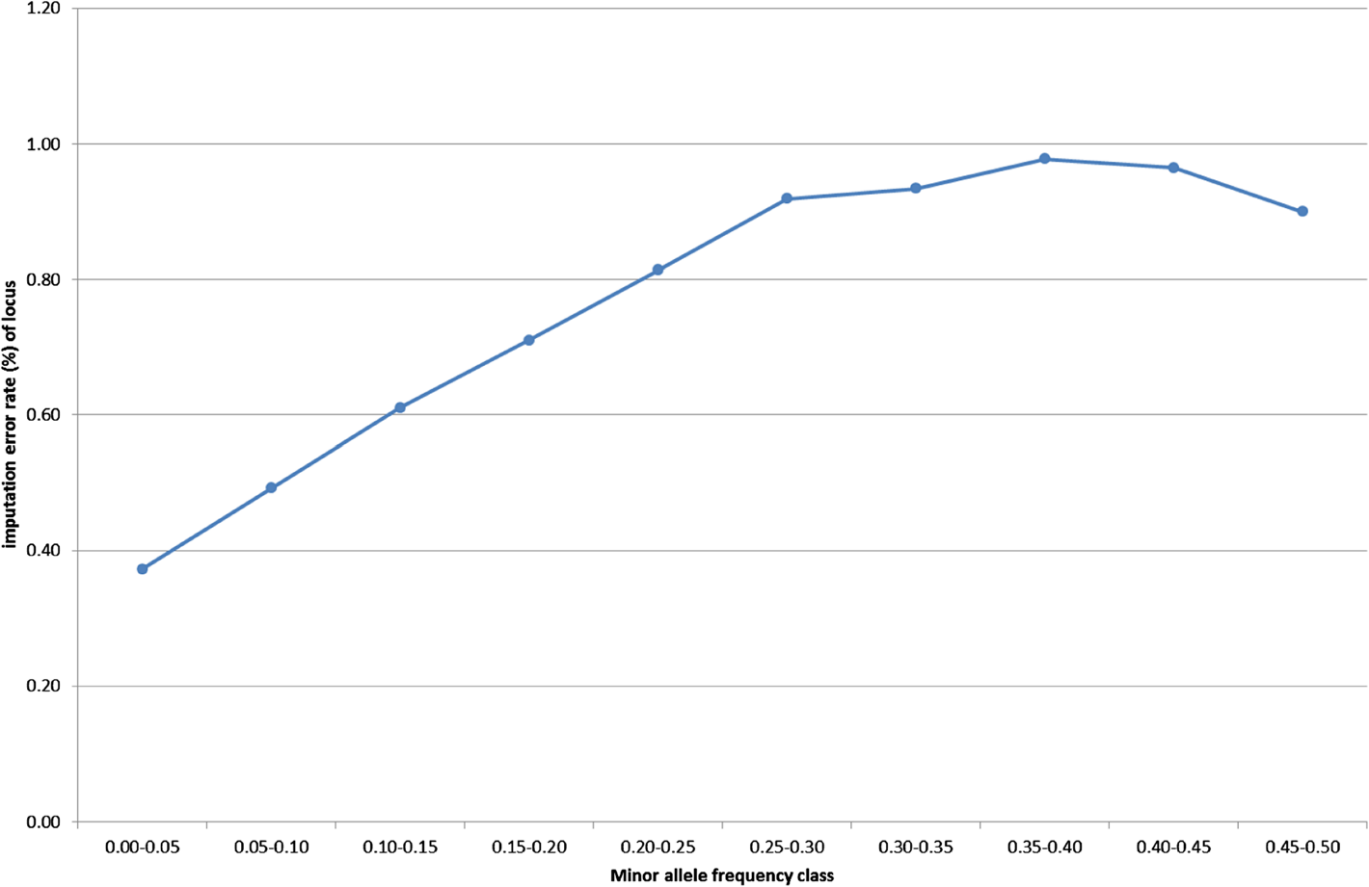
**Average locus imputation error rate (%) for each of 10 classes of SNPs according to their minor allele frequency.** Results are presented for the combination of method C (Beagle version 3.3.0) and dataset 2 (1289 HD-genotyped animals).

Figure 2 shows the allelic imputation error rate for each of 20 classes of allele frequency, for four combinations of method and dataset: method A, B, and C applied to dataset 1, and method C applied to dataset 2. For all four alternatives, the highest imputation error rate was found for alleles with the lowest frequency, ranging from 3.1% for method C applied to dataset 2, to 17.8% for method A applied to dataset 1. For allele frequencies higher than 0.50, the imputation error rate was less than 1%, except for method A applied to dataset 1, for which the imputation error rate was less than 1% only for classes of allele frequency larger than 0.80. When results are evaluated on a per-locus base, then the low frequency alleles contribute relatively little to the overall imputation error rate. The low frequency allele is always associated with a high frequency allele which has a low imputation error rate, as shown in Figure 3, in which results are presented as the imputation error rate per minor allele frequency class, for the analysis of method C (Beagle version 3.3.0) on dataset 2 (1289 HD-genotyped animals). The highest imputation error rate was observed for the class of SNPs with a minor allele frequency between 0.35 and 0.40, and the lowest imputation error rate for SNPs with a minor allele frequency between 0.00 and 0.05.

**Figure 4:**
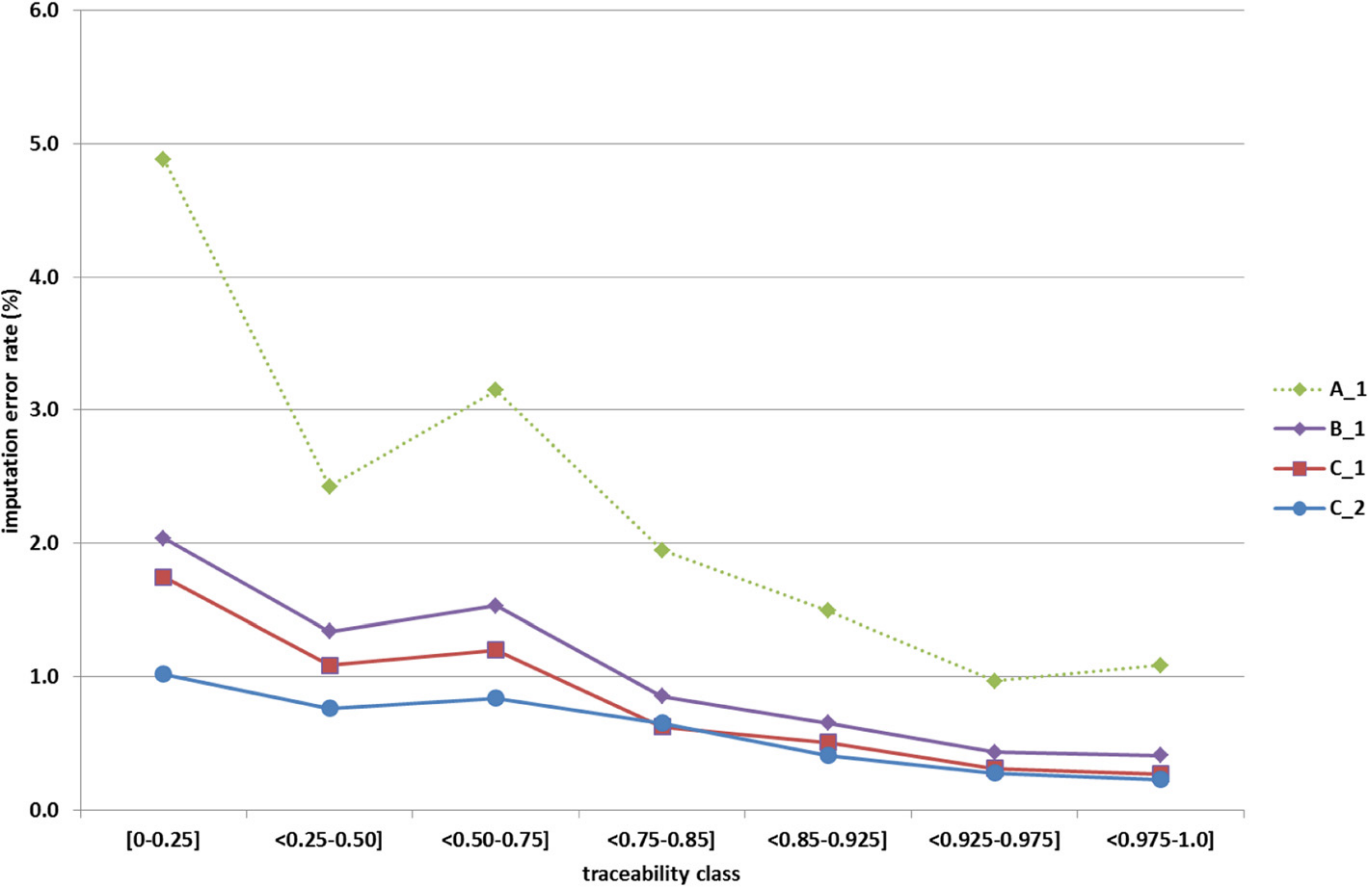
**Average allelic imputation error rate (%) for each of seven traceability classes.** Results are presented for four combinations of method (A, B or C) and dataset (1 or 2). Method A: combination of Beagle 2.1.3 and DAGPHASE with scale and shift parameters equal to 2.0 and 0.1; method B: same as method A, but with scale and shift parameters equal to 1.0 and 0.0; method C: Beagle version 3.3.0; dataset 1: 548 HD-genotyped animals; dataset 2: 1289 HD-genotyped animals.

Figure 4 shows imputation error rates for each of a number of traceability classes. Imputation error rates were lower with higher traceability, i.e. when more HD-genotyped ancestors are present in the dataset. Results are presented for four combinations of method and dataset: method A, B, and C applied to dataset 1, and method C applied to dataset 2. For classes of animals with traceability above 0.50, there was a clear decrease in the imputation error rate with increasing traceability.

Table 4 shows the average, minimum and maximum time needed per imputation method in dataset 1, calculated across all analyzed chromosomes and replicates. For this dataset, computation time was lowest for method D, DAGPHASE using the DAG obtained from Beagle. Computation time was highest for method B, DAGPHASE/Beagle with scale and shift parameters equal to 1.0 and 0.0, respectively.

**Table 4:** Average, minimum and maximum computation time (h) per chromosome for each of four methods applied to dataset 1

		**Method**		
	**A**	**B**	**C**	**D**
Average	1.7	6.9	0.7	0.3
Minimum	0.8	3.0	0.4	0.2
Maximum	2.9	11.9	1.1	0.5

Method A is a combination of Beagle 2.1.3 and DAGPHASE with scale and shift parameters equal to 2.0 and 0.1; method B is the same as method A, but with scale and shift parameters equal to 1.0 and 0.0; method C is Beagle version 3.3.0; method D is DAGPHASE using DAG from method C.

## Discussion

Imputation is a useful tool to obtain high-density genotypes for animals of interest while genotyping only part of the animals with the high-density chip. The remaining subset of the animals can be genotyped on lower density chips. However, there are errors associated with imputation, and some of the factors that influence imputation error rates were studied. Imputation from a moderate-density chip (50 k) to a high-density chip (777 k) was analyzed in three different datasets, to study the influence of both size and composition of the reference population for imputation on imputation error rates.

### Size of the reference population

In datasets 1 and 2, with 488 and 1229 reference animals respectively, all reference animals were genotyped with the HD-chip. Beagle 3.3.0 was used to compare results from these two datasets. The average imputation error rate decreased from 0.67% for dataset 1 (the smallest dataset) to 0.41% for dataset 2. This result shows that, although the imputation error rate was already small in dataset 1, the imputation error rate could be further decreased by adding more animals genotyped on the HD-chip to the reference set. Although intermediate sizes of the reference population were not tested, it is expected that the effect of further increasing the size of the reference population is limited. This expectation is based on analysis of subsets of dataset 1; when size of the reference population was increased from 200 to 300, 400, and 500, the imputation error rate decreased by 0.17, 0.13 and 0.04%, respectively.

### Imputation method parameterization

Datasets 1 and 3 were analyzed with various imputation software packages. In this study, Beagle performed better than the combination of Beagle and DAGPHASE (Table 3). Using DAGPHASE has an advantage when linkage information is important, e.g. when both parents are genotyped or when the dataset contains large families. There is no advantage to using DAGPHASE when the density of the lower density chip is already relatively high, as in our study, because linkage disequilibrium information is more accurate at higher densities and can be extracted by Beagle with greater accuracy than by DAGPHASE. This study also showed that the parameters used for the DAGPHASE/Beagle combination strongly influenced imputation error rates. When scale and shift parameters were equal to 1.0 and 0.0, respectively, instead of 2.0 and 0.1, imputation error rates decreased from 1.91 to 0.87% (Table 3, method A and B), at the expense of longer computation times (Table 4). It is expected that lower values for the scale and shift parameters will result in lower imputation error rates in most datasets, but the balance between decreasing imputation errors and longer computation times may lead to different optimal parameters in different datasets.

### Chromosome characteristics

Sun et al. [19] observed that imputation accuracy was positively associated with chromosome size, although this was based on an analysis of three chromosomes covering the range of chromosome sizes. In our study, chromosomes ranked differently for imputation error rates across the different datasets and methods studied (Table 3). In most dataset-method combinations, BTA14 and BTA6 had the lowest imputation error rates, although BTA1 was the largest chromosome. In all dataset-method combinations, BTA29 had the highest imputation error rate. BTA14 had the highest number of BovineSNP50 markers per cM, and BTA29 had the lowest number of BovineSNP50 markers per cM (n50k/cM, Table 2). Based on the characteristics mentioned in Table 2, the number of BovineSNP50 markers per cM influenced imputation error rate more than chromosome size.

### Allele frequency

Although imputation errors were low on average, they can be quite high for individual alleles. This is illustrated in Figure 2: on average, low frequency alleles had relatively high imputation error rates. Because the low frequency alleles are not represented in any reference haplotype, the imputation program cannot derive the correct allele. The impact of errors for these alleles on the total imputation error rate is relatively low, because of their low frequency (Figure 3). However, if these low frequency alleles are associated with deleterious alleles, then errors in imputation may have an impact on results of association studies for genetic defects, for example.

### Marker map

Not only allele frequency, but also errors in the marker map can cause high imputation error rates for particular loci. Erbe et al. [20] studied imputation to the HD-chip and found more than 20% incorrectly imputed genotypes for each SNP in a subset of 1231 SNPs. When these SNPs were remapped, imputation error rates were substantially lower for 601 of the 1231 SNPs. This factor may play a role in our study, because the map we used was the same as that used by Erbe et al. [20]. This means that imputation error rates could be even lower if a more accurate map became available.

### Relationship between the imputed animal and the reference population

Figure 4 shows that the imputation error rate depends on the relationship between the imputed animal and the reference set used for imputation, as measured by traceability. When constructing a reference population for imputation to higher density chips or platforms (e.g. whole-genome sequence), the policy to include the closest ancestors will contribute to a decrease in imputation errors.

### Other studies

A few studies have investigated imputation from 50 k to HD chips. Larmer et al. (2012, unpublished data) studied imputation in the Holstein, Guernsey and Ayrshire breeds. The percentage of incorrect genotypes in the Holstein breed using 892 reference animals for imputation was 0.70%, which corresponds to an allelic imputation error rate of approximately 0.35%, which is slightly lower than in our study. Adding the two other breeds to the reference set did not decrease the error rate. Erbe et al. [20] reported a genotype imputation error rate of 2.3% when using approximately 450 heifers as reference to impute 450 other heifers from the 50 k to the HD chip. When 93 key ancestor bulls were added to the reference population, the genotype imputation error rate decreased to 2.0%. In the Jersey breed, with only 93 key ancestors in the reference set, the genotype imputation error rate was equal to 4.2%. Allelic imputation error rates can be considered as equal to half of these values. Brøndum et al. [21] showed that increasing the size of the reference population used for imputation by adding animals from other breeds is useful only if the breeds are related. Their study investigated imputation from 50 k to HD in Nordic Red breeds using different combinations of Nordic Red and Holstein animals in the reference population. With a reference set of 556 animals, consisting of animals of three breeds, the allelic imputation error rate was equal to 0.96% for 150 animals.

### Implications

This study investigated imputation errors from the 50 k to the HD chip. However, many animals are and will be genotyped with lower density panels, especially the Illumina BovineLD panel (6.9 k). As shown by Larmer et al. (2012, unpublished data), imputation error rates when using FImpute [6] were lower when imputation was first from the BovineLD to the 50 k chip and subsequently from the 50 k to the HD chip, rather than imputing directly from the BovineLD to the BovineHD chip. This may influence the implementation of imputation from various lower density chips to HD chips in routine operations.

Mulder et al. [15] analyzed the impact of imputation errors on the reliability of GEBV. The average imputation error rate was 3.8% for imputation from a 3 k in silico chip to a 50 k chip. Reliability of GEBV based on imputed genotypes was 2.1% lower than the reliability of GEBV based on real genotypes, averaged across 10 traits. For imputation from a 6 k in silico chip to a 50 k chip, the average imputation error rate was 2.6%. Reliability of GEBV based on imputed genotypes was 1.2% lower than the reliability of GEBV based on real genotypes. In French and Nordic data, the reliability of GEBV with imputed genotypes was 2 to 6% lower than the reliability of GEBV with real genotypes, for imputation from the 3 k to the 50 k chip [14]. In our study, we investigated the error rate for imputation from 50 k to HD chips. The impact of imputation error rate on the reliability of GEBV is expected to be relatively small, because the imputation error rate was small (0.41%).

When imputation from lower density chips to HD chips is part of a weekly or monthly procedure for routinely processing genotypes, computing time may be an important factor in the choice of the imputation method. In this study, using Beagle only (method C) gave the lowest imputation error rates, and was relatively fast. However, computing time using Beagle increased relatively quickly when the number of animals genotyped with a moderate-density chip increased, compared to using a combination of DAGPHASE and Beagle (methods A and B), or with DAGPHASE using the DAG obtained from Beagle (method D) (results not shown). Because imputation error rates with method D were only slightly higher than imputation error rates with method C, method D is an attractive alternative for routine procedures, once the DAG from method C is obtained.

## Conclusions

The allelic imputation error rate for imputation from the Illumina BovineSNP50 to the Illumina BovineHD chip was equal to 0.67% when a reference set of 488 animals was used. This imputation error rate was reduced to 0.41% when the reference set was increased to 1229 animals. In both situations, Beagle 3.3.0 was used. Results were slightly improved by adding animals genotyped with the 50 k chip to the dataset. Application of DAGPHASE using information from Beagle gave slightly poorer results but is an interesting alternative in applications where computing time is a limiting factor.

## Competing interests

The authors declare that they have no competing interests.

## Authors’ contributions

CS, VD, MSL, ZL and TD participated in the design of the study. CS, RD, RFB, JC and ZL contributed to the data analysis. CS drafted the manuscript, refined by RFB, VD, MSL, OGR, JP and TD. All authors read and approved the final manuscript.
